# Potential distribution of *Notopterygium incisum* Ting ex H. T. Chang and its predicted responses to climate change based on a comprehensive habitat suitability model

**DOI:** 10.1002/ece3.6117

**Published:** 2020-03-05

**Authors:** Zefang Zhao, Yanlong Guo, Haiyan Wei, Qiao Ran, Jing Liu, Quanzhong Zhang, Wei Gu

**Affiliations:** ^1^ School of Geography and Tourism Shaanxi Normal University Xi'an China; ^2^ Faculty of Geographical Science Beijing Normal University Beijing China; ^3^ National Engineering Laboratory for Resource Development of Endangered Crude Drugs in Northwest China Shaanxi Normal University Xi'an China; ^4^ National Tibetan Plateau Data Centre Institute of Tibetan Plateau Research Chinese Academy of Sciences Beijing China; ^5^ The Key Laboratory of Medicinal Resources and Natural Pharmaceutical Chemistry The Ministry of Education Shaanxi Normal University Xi'an China; ^6^ College of Life Sciences Shaanxi Normal University Xi'an China

**Keywords:** climate change, ensemble model, *Notopterygium incisum* Ting ex H. T. Chang, potential distribution, species distribution models

## Abstract

*Notopterygium incisum* Ting ex H. T. Chang is a rare and endangered traditional Chinese medicinal plant. In this research, we built a comprehensive habitat suitability (CHS) model to analyze the potential suitable habitat distribution of this species in the present and future in China. First, using nine different algorithms, we built an ensemble model to explore the possible impacts of climate change on the habitat distribution of this species. Then, based on this model, we built a CHS model to further identify the distribution characteristics of *N. incisum*‐suitable habitats in three time periods (current, 2050s, and 2070s) while considering the effects of soil and vegetation conditions. The results indicated that the current suitable habitat for *N. incisum* covers approximately 83.76 × 10^3^ km^2^, and these locations were concentrated in the Tibet Autonomous Region, Gansu Province, Qinghai Province, and Sichuan Province. In the future, the areas of suitable habitat for *N. incisum* would significantly decrease and would be 69.53 × 10^3^ km^2^ and 60.21 × 10^3^ km^2^ in the 2050s and 2070s, respectively. However, the area of marginally suitable habitat would remain relatively stable. This study provides a more reliable and comprehensive method for modelling the current and future distributions of *N. incisum*, and it provides valuable insights for highlighting priority areas for medicinal plant conservation and resource utilization.

## Graphical abstract

Built a comprehensive habitat suitability model (CHS) for *Notopterygium incisum*, which constructed separately for the climate, vegetation, and soil environment demands. Climate change will intensify the destruction and fragmentation of native habitats of *N. incisum*, which will lead to the disappearance of suitable habitat in these areas, increasing the risk of the extinction of this species.

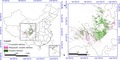

## 1 INTRODUCTION

The growth, development, and reproduction of plants are limited by climatic and other environmental factors. Global warming is likely to result in changes in biological habitats, losses of regional species diversity, and increases in the risk of species extinction (Anderson, 2013; Marzloff et al., 2018; Pacifici et al., 2015; Urban, 2015). Topography affects the redistribution of moisture and heat in the natural environment; therefore, topography is an important driver of plant species distributions, especially for alpine plants, and in mountainous regions, macrotopographies are usually large enough to provide refuge for plant species under changing climates (Myan, Walker, & Paramor, 2013). The Qinghai–Tibet Plateau uplifted more than 3,000 m in the Quaternary period, which dramatically changed the topography and climate in this region and formed various climate types (Zhang, Fengquan, & Jianmin, 2000). Meanwhile, microtopography will hinder the ability of a species to shift poleward and upslope because it can cause a relatively stable and closed climate environment within a short distance (Patsiou, Conti, Zimmermann, Theodoridis, & Randin, 2015). Hence, microtopography created a biological refuge for many rare, relict, and endemic plants during the last glacial maximum (Elsen & Tingley, 2015; Yang, Zhou, Li, Song, & Chen, 2011). Global warming will inevitably affect the living environment and hydrothermal conditions of plants in the Qinghai–Tibet Plateau region, and the complex topography will exacerbate this impact (Li et al., 2013).

Models are useful tools for simulating the impact of future climate change on plant species distribution, especially in this study, which considered a large spatiotemporal scale. In recent years, species distribution models (SDMs) have been popular tools for assessing the spatial–temporal variations in species distributions under different climate scenarios (Anderson, 2013; Mcquillan & Rice, 2015; Zhang et al., 2014). In the past two decades, advancements in statistical methods have promoted the development of SDMs, and numerous statistical methods and software programs have been developed to describe the niche characteristics of species and predict species distribution patterns. The popular algorithms are as follows: surface range envelope (SRE, i.e., BIOCLIM) (Booth, Nix, Busby, & Hutchinson, 2014), flexible discriminant analysis (FDA) (Basile et al., 2016), generalized linear model (GLM) (Lopatin, Dolos, Hernández, Galleguillos, & Fassnacht, 2016), generalized additive model (GAM) (Muñoz‐Mas, Papadaki, Martinez‐Capel, Zogaris, & Ntoanidis, 2016), multiple adaptive regression splines (MARS) (Friedman, 1991), generalized boosting model (GBM) (Moisen et al., 2006), classification tree analysis (CTA) (Thuiller & Lavorel, 2010), artificial neural network (ANN) (Segurado & Araujo, 2004), random forest (RF) (Mi, Huettmann, Guo, Han, & Wen, 2017), and maximum entropy (MaxEnt) (Phillips, Anderson, & Schapire, 2006). However, differential niche requirements of species shape the geographic distribution of species within an environment. Hence, the following factors should be considered when selecting an appropriate SDM algorithm for species distribution research on specific spatiotemporal scales, that is, species niche characteristics, especially those related to specific traits of species; environmental characteristics, especially the limiting factors in species' habitats; and data quality, including data availability and the spatial and temporal resolution of data (Bell & Schlaepfer, 2016; Guisan, Thuiller, & Zimmermann, 2017; Li & Wang, 2013). Simultaneously, for the same species with the same input data, different response curves and variable weights can be adopted according to the corresponding statistical properties of different algorithms, which will lead to different simulation results (Guo, Li, Zhao, & Wei, 2018) and will notably increase the uncertainties in the predicted species distribution. Therefore, the ensemble model (EM) strategy is proposed to solve this problem. This strategy applies several SDM methods within a consensus modelling framework and reduces the predictive uncertainty of individual models by combining the results from multiple models (Araújo & New, 2007; Guisan et al., 2017). Previous studies have indicated that EMs can substantially improve model accuracy and applicability (Grenouillet, Buisson, Casajus, & Lek, 2011).

*Notopterygium incisum* Ting ex H. T. Chang, also known as the “Emissary of Qiang Nationality” and “messenger of King Hu,” is a Chinese herbal medicine associated with many beautiful and moving folk stories, and it is also an endangered traditional Tibetan medicinal plant (Committee of Flora of China, 1986). According to field investigations and the literature, *N. incisum* is mainly distributed in Shaanxi Province, Sichuan Province, Qinghai Province, and the Tibet Autonomous Region (Committee of Flora of China, 1986). Modern science has confirmed that *N. incisum* can be used to relieve inflammation, cure arrhythmia and myocardial ischemia, promote cerebral circulation, remove thrombosis and bacteria, and carry out other pharmacological actions (Committee of National Pharmacopoeia, 2015; Li, Zhang, Wang, & Lei, 2003). Due to the increasing demands in the domestic and international medical markets and increasingly intense human activities, *N. incisum* is becoming increasingly threatened, primarily due to habitat disturbance and destruction (Liu, 2006; Sun et al., 2015; Zhou et al., 2003). In addition, this plant has been included on the Red List of endangered species in China since 2005. Thus, information on the potential geographic distribution and its response to climate change is vital to the protection and resource utilization of *N. incisum*.

Existing studies on *N. incisum* mainly focus on the identification of medicinal components, pharmacological analyses, cultivation, industrialization, and resource investigations (Gao & Fang, 2007; Li, Zhou, & Zhou, 2011; Li et al., 2003; Peng, Dong, Zhu, & Yan, 2006), while the emphasis of our research is on the distribution of *N. incisum*. This research analyzed the potential habitat distribution characteristics of *N. incisum* under future climate change scenarios, exploring the relationship between the spatial and temporal distribution of *N. incisum* and related geographic environmental factors. Additionally, this research will help with the introduction and cultivation of a good agricultural practice (GAP) and germplasm resource repositories under different environmental change conditions.

## 2 MATERIALS AND METHODS

### 2.1 Occurrence data

**Figure 1:**
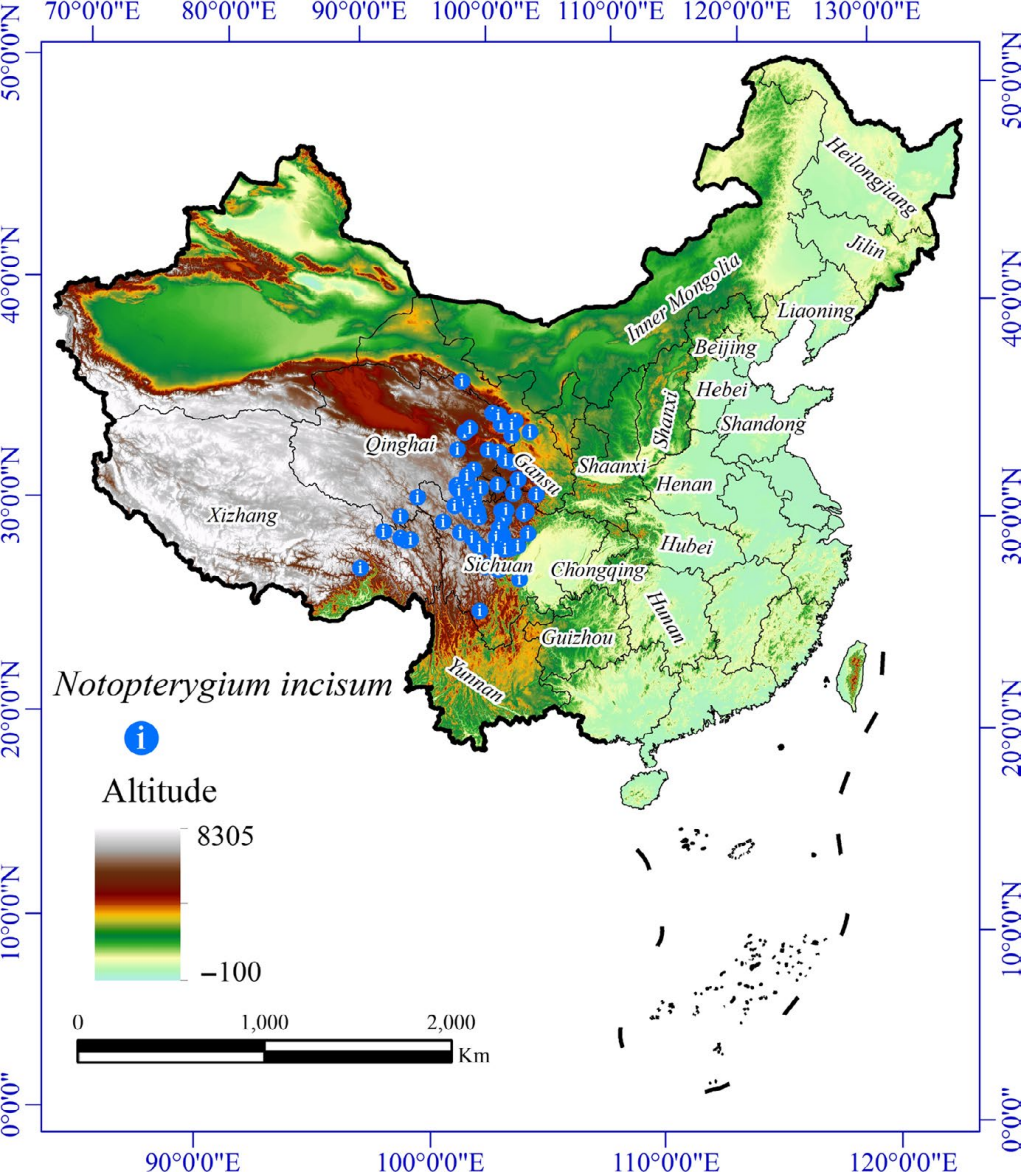
Study area and geographic locations of *Notopterygium incisum* population distributions

In this research, the occurrence data for this species were from a variety of sources, including published scientific literature, field survey reports (Jiang et al., 2006; Liu, 2006; Sun et al., 2015), and online plant databases, such as the Global Biodiversity Information Facility (GBIF, 2019) and Chinese Virtual Herbarium (CVH, 2019). To improve the geographic accuracy and perform scientific modelling, we selected only data with precise longitude and latitude information and removed duplicate coordinates and incomplete information. Finally, we obtained 99 occurrence data points to build the model (Figure 1, Table S1).

### 2.2 Pseudo‐absence data

**Figure 2:**
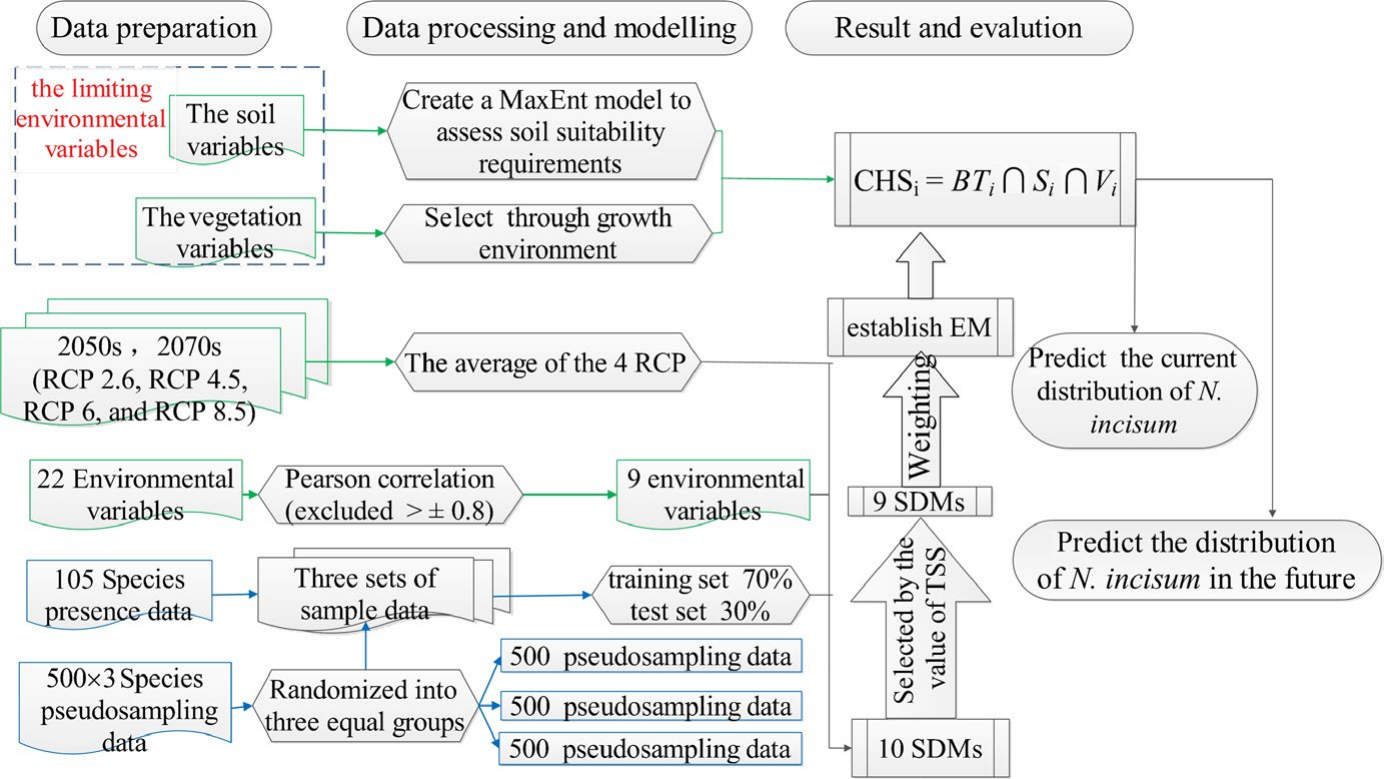
Modelling process in a flow diagram

In practice, most SDM models require species absence data; however, true absence data are usually not available, so we use pseudo‐absence data as a substitute (Zhang et al., 2016). The numbers and qualities of pseudo‐absence data affect the accuracy of the SDMs (Zhang et al., 2016). In this research, using the “Sample by Buffered Local Adaptive Convex‐Hull” tool in the SDMtoolbox 2.0 (Brown, Bennett, & French, 2017), we generated pseudo‐absence data (Barbet‐Massin, Jiguet, Albert, & Thuiller, 2012) within 200 km buffers around the occurrence points. We selected three random subsets of the background to generate three groups of pseudo‐absence data, and each group included 500 points (Figure 2).

### 2.3 Environmental variables

In this study, we chose four categories of environmental datasets with a total of 24 environmental variables to characterize the environmental demands of *N. incisum* (Table 1). Among these environmental variables, all climate variables, including 19 bioclimatic variables under three time periods, were downloaded from the WorldClim dataset (WORLDCLIM, 2019), with a resolution of 30″ (approximately 1 km^2^) (Fick & Hijmans, 2017). The future time periods were set to the 2050s (average for 2041–2060) and 2070s (average for 2061–2080), and we used bioclimatic variables under four IPCC–CMIP5 representative concentration pathways (RCPs), namely, RCP2.6, RCP4.5, RCP6, and RCP8.5, which are labelled after the possible range of radiative forcing values in the year 2100 (2.6, 4.5, 6.0, and 8.5 W/m^2^, respectively) (IPCC, 2013) to depict future temperature and rainfall. We used bioclimatic variables from three GCMs (BCC–CSM1–1, MIROC5, and CCSM4) for model building.

**Table 1:** Environmental element index used for predicting the potential geographic distribution of *Notopterygium incisum*

Environmental index	Code	Name	Selection
Climate variables	Bio1	Annual mean air temperature	√
	Bio2	Mean diurnal temperature range	
	Bio3	Isothermality	
	Bio4	Temperature seasonality	√
	Bio5	Max temperature of warmest month	
	Bio6	Min temperature of coldest month	
	Bio7	Temperature annual range	
	Bio8	Mean temperature of wettest quarter	
	Bio9	Mean temperature of driest quarter	
	Bio10	Mean temperature of warmest quarter	√
	Bio11	Mean temperature of coldest quarter	√
	Bio12	Annual precipitation	√
	Bio13	Precipitation of wettest month	
	Bio14	Precipitation of driest month	
	Bio15	Precipitation seasonality	√
	Bio16	Precipitation of wettest quarter	
	Bio17	Precipitation of driest quarter	
	Bio18	Precipitation of warmest quarter	
	Bio19	Precipitation of coldest quarter	√
Topographic variables	ASL	Elevation above sea level	
	SLOP	Slope	√
	ASPE	Aspect	√
Soil variable	ST	Soil type variables	√
Vegetation variable	VT	Vegetation type variables.	√

Note

√ means the variables were selected to build the model, for a total of 11 variables.

The topographic variables, including elevation, slope, and aspect, have the same resolution as the bioclimatic variables. The elevation variable was also acquired from the WorldClim dataset, and the slope and aspect variables were generated by the ArcGIS spatial analysis function based on the elevation variable.

In this research, the 1:1 million soil database of China was used for the soil type variables, and the 1:1 million China vegetation data were used for the vegetation type variables. Both of these data types were acquired from the National Tibetan Plateau Data Centre (Li, Nan, et al., 2011; NTPDC, 2019; Ran, Li, Lu, & Li, 2012) and had the same resolution as the other environmental variables. Because changes in vegetation and soil types lag behind climate change (Wu et al., 2015), we used the same soil type and vegetation type data in the future climate change scenarios.

### 2.4 Methods

The “biomod2” package is an object‐oriented, expandable, and reproducible R platform for forecasting species distributions (R Project, 2018; Thuiller, Lafourcade, Engler, & Miguel, 2009). The “biomod2” package supports 10 model algorithms (i.e., ANN, CTA, FDA, GAM, GBM, GLM, MaxEnt, MARS, RF, and SRE), and in this study, we separately used all 10 model algorithms to build the corresponding SDM. The accuracy of each model algorithm was evaluated by the area under the receiver operating characteristics (ROC) curve (AUC) and true skill statistic (TSS) (Thuiller et al., 2009). Then, we selected the algorithm that met the precision requirements to construct an EM to simulate the migration trend of *N. incisum*. Finally, we used biotic variables (vegetation and soil) to further refine the EM results.

#### 2.4.1 Input data processing

In this research, the processing of input data included the removal of collinearity between environmental variables and the generation of pseudosampling points. First, principal component analysis (PCA) (Supplementary materials 1) and Pearson correlations were used to select a subset of environmental variables (Guisan et al., 2017; Guo, Li, Zhao, & Nawaz, 2019). Finally, we selected seven bioclimatic variables and two topographic variables (i.e., annual mean air temperature, bio1; temperature seasonality, bio4; mean temperature of the warmest quarter, bio10; mean temperature of the coldest quarter, bio11; annual precipitation, bio12; precipitation seasonality, bio15; precipitation of the coldest quarter, bio19; slope; and aspect) (Table 1). Second, three sets of pseudosampling points were randomly generated, and there were 500 pseudosampling points in each group. We obtained three sets of model inputs using the three sets of pseudosampling points and the *N. incisum* occurrence data.

#### 2.4.2 Single modelling technique

We separately input the three sets of data into the model. For each modelling process, the sampling data of the *N. incisum* points involved in the modelling were divided into two parts. Seventy percent of the sampling data were used as a training set, and the remaining data were used as testing data. In addition, we set an equal weight for the total presence of sampling points and pseudosampling points. We used TSS and AUC to evaluate the model, and Equation (1) for TSS is as follows:

(1) TSS=Sensitivity+Specificity-1

(2) Sensitivity=a/(a+c)

(3) Specificity=d/(b+d)

where *a* refers to the number of true positives, *b* refers to the number of false positives, *c* refers to the number of false negatives, and *d* refers to the number of true negatives. TSS values > 0.6 are considered good (Allouche, Tsoar, & Kadmon, 2006; Jones, Acker, & Halpern, 2010). The AUC indicates the area under the ROC curve, and the ROC plots display the relationship between sensitivity (Equation (2)) and 1—specificity (Equation (3)) over a range of threshold values (0–100). For AUC, which does not require selection of a habitat suitability threshold, values > 0.9 are considered good, and values from 0.7 to 0.9 are considered moderate (Allouche et al., 2006; Jones et al., 2010).

In addition, for each modelling algorithm, we repeated this modelling process 10 times with a bootstrapping sampling strategy; thus, in total, we built 300 single models (three sets of sampling data × 10 single modelling techniques × 10 repeats).

#### 2.4.3 Ensemble model

Here, we used an EM to reduce the uncertainty caused by different modelling algorithms and sample datasets (Ran et al., 2012; Zhao, Wei, Guo, & Gu, 2016). First, we excluded the SRE because the average TSS value of this method was <0.5. Second, and we used the TSS values to determine the weight of the other 270 single model results. Equation (4) is as follows:

(4) $$w_{j}=\frac{r_{j}}{\sum_{j=1}^{h}r_{j}}$$

where *w_j_* refers to the weight of the results of model *j*, *r_j_* refers to the TSS of model *j*, and *h* (*h* = 270) refers to the number of model results.

Then, we constructed the EM using Equation (5):

(5) $${\text{BT}}_{i}=\sum_{j=1}^{n}w_{j}\times x_{\mathrm{ij}}$$

where *BT_i_* refers to the potential habitat suitability index of the evaluation unit (grid) *i*, *w_j_* refers to the weight of the results of model *j*, and *x_ij_* refers to the value of evaluation unit *i* in the results of model *j*. The comprehensive evaluation index for the potentially suitable habitat distribution of *N. incisum* is *BT_i_* (range [0,1]). When the value of *BT_i_* is close to 1, the geographic space of the grid unit is considered suitable for the growth of *N. incisum*.

#### 2.4.4 Comprehensive habitat suitability model

In its natural habitats, *N. incisum* is not the dominant species and is usually an associated species that grows in alpine shrublands, alpine meadows, and woodlands (Mcquillan & Rice, 2015). Hence, the vegetation types are the important limiting factors, reflecting the limitations of the species' migration ability, and here, according to the literature, we conducted binary conversion of the vegetation type data. A suitability value of 1 was defined as a suitable vegetation type for *N. incisum*, namely, alpine forest land, shrubland, and alpine meadows, while all other vegetation types received a value of 0. Because there are no clearly defined soil types that are suitable for the growth of *N. incisum*, we used the soil type variables and occurrence data of *N. incisum* to create a MaxEnt model to assess the soil suitability requirements. The model settings were as follows: 75% of the occurrence data were used as a training set, and the remaining data were used as testing data. Ten replications were performed, and the AUC was used to evaluate model performance.

Then, based on the EM and considering the effect of soil and vegetation conditions, we built a CHS model to further identify the distribution characteristics of *N. incisum*‐suitable habitats, and the index of CHS for *N. incisum* was defined using the following equation (Equation (6)):

(6) $${\text{CHS}}_{i}={\text{BT}}_{i}\cap S_{i}\cap V_{i}$$

where *CHS_i_* refers to the comprehensive habitat suitability index for *N. incisum* in evaluation unit *i*, *BT_i_* refers to the value of the EM result in evaluation unit *i*, *S_i_* refers to the result of soil suitability in evaluation unit *i*, and *V_i_* refers to the habitat suitability value for vegetation. Here, we classified the whole result into three classes based on the *BT_i_*, *S_i_*, and *V_i_* values: unsuitable habitats, *BT_i_* or *S_i_* values below 0.3 (i.e., *BT_i_* < 0.3 or *S_i_* < 0.3); marginally suitable habitats, with a *V_i_* value of 1, *BT_i_* and *S_i_* values ≥ 0.3, but at last one of them below 0.5 (i.e., (0.3 ≤ *BT_i_* < 0.5 and *S_i_* ≥ 0.3) or (0.3 ≤ *S_i_* < 0.5 and *BT_i_* ≥ 0.3)); and suitable habitats, *BT_i_* and *S_i_* values ≥ 0.5 (i.e., *BT_i_* ≥ 0.5 and *S_i_* ≥ 0.5).

**Figure 3:**
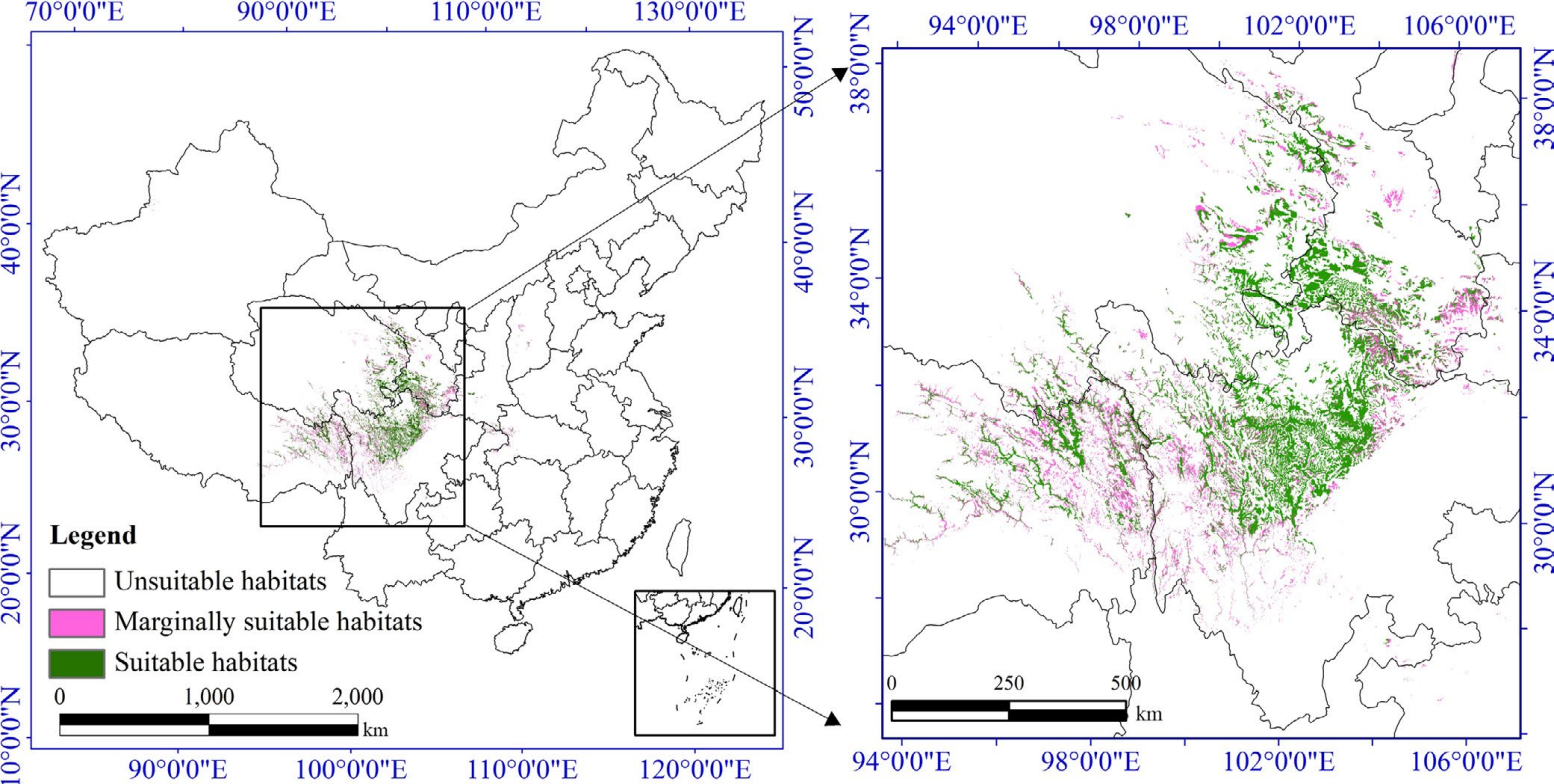
Potential habitat distribution of *Notopterygium incisum* predicted by the ensemble model at present

**Figure 4:**
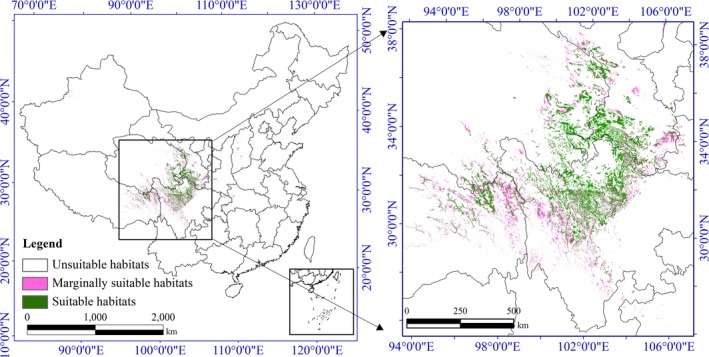
Potential spatial distribution of *Notopterygium incisum* based on the ensemble model in the 2050s

**Figure 5:**
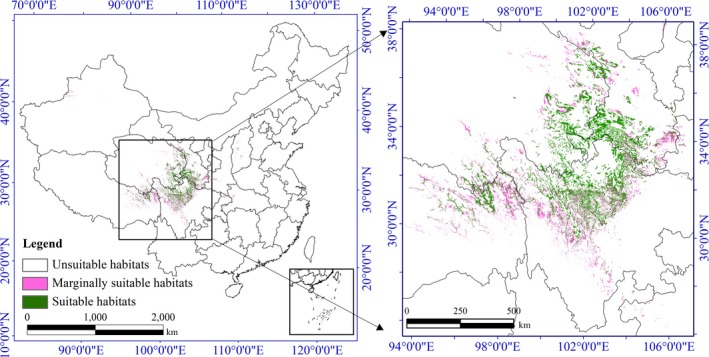
Potential spatial distribution of *Notopterygium incisum* based on the ensemble model in the 2070s

Finally, we used the CHS model to simulate the potential changes in the suitable habitat distribution for *N. incisum* in the future (Figure 2). Based on bioclimatic variable data from the three GCMs of two future periods (2050s and 2070s), we used the EM to explore the possible impacts of climate change on the habitat distribution of this species. The future EM results for *N. incisum* under each IPCC–CMIP5 RCP and for both periods were obtained by averaging the results. We used the average result of the four IPCC–CMIP5 RCP scenarios as the final EM result. Then, combined with the same result of soil suitability, we obtained the future potential suitable habitat distribution with the CHS model. At last, we mapped all habitat suitability values of *N. incisum* for three periods (current, 2050s, and 2070s) and calculated the areas in Figures 3, 4, 5.

## 3 RESULTS

### 3.1 Model performance

**Figure 6:**
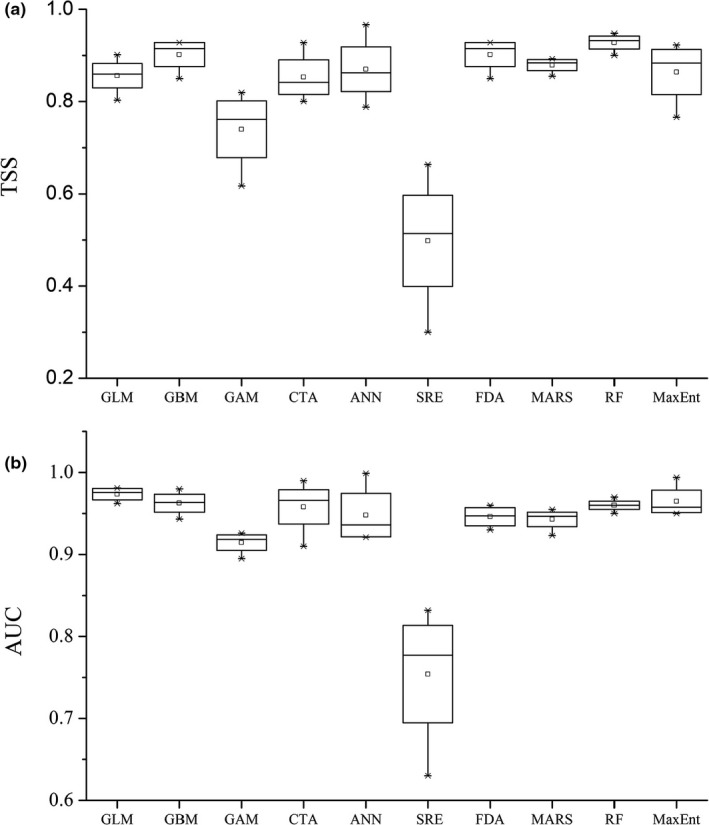
Boxplots of the predictive performance of the different techniques used for SDMs. (a) TSS and (b) AUC

The statistical accuracy results for the 10 models showed that RF was the best model, and the TSS and AUC values were 0.929 and 0.984, respectively. This model was followed by FDA and GBM, and the TSS values for these two models were above 0.90. The accuracy of the SRE was lowest, and the TSS and AUC values were 0.50 and 0.75, respectively (Figure 6). In addition, in the nine models involved in the EM, the average TSS value of each single model was >0.74, and the average AUC value of each single model was >0.91 (Figure 6). Therefore, the EM provided satisfactory results, and the TSS and AUC values were 0.80 and 0.96, respectively. The AUC value of the MaxEnt model for the soil environment requirements was 0.87, which means that the model results were scientific and reasonable.

### 3.2 Distribution of suitable habitats in the current climate environment

In this study, different statistical algorithms led to different simulation results, but the spatial distribution patterns of *N. incisum* were consistent. In addition, all model results showed that the suitable distribution area for *N. incisum* was concentrated in the transitional zone from the first step to the second step in the “ladder topography” of western China, including the edge of the Qinghai–Tibet Plateau and the western Sichuan Plateau.

Based on the prediction results of the EM, we mapped the distribution of suitable habitats for *N. incisum* in China (Figure 3). The results showed that suitable habitats for *N. incisum* were mainly distributed in the region along the Qilian Mountains, northwest of the Qinling‐Daba mountain area in Gansu Province, eastern margin of the Tibetan Plateau and a great portion of the western Sichuan Plateau. The marginally suitable habitats were distributed in some areas of Gansu Province, east of Qinghai Province, southeast of Tibet Autonomous Region, west and north of Sichuan Province, part of southwest Shaanxi Province, part of Chongqing municipality, Shennongjia Forestry District of Hubei Province, Shanxi Province, Xinjiang Uygur Autonomous Region, Guizhou, and Yunnan Province, which had a sporadic distribution. In addition, the habitats of *N. incisum* were fragmented with large local patches of suitable habitat that were not spatially contiguous. We also calculated the areas of suitable habitats and marginally suitable habitats for *N. incisum*, and the results showed that the suitable habitats had an area of approximately 83.76 × 10^3^ km^2^, and the marginally suitable habitats had an area of approximately 102.72 × 10^3^ km^2^ (Table 2). In summary, the proportions of *N. incisum*‐suitable habitats were small and narrow, and the distribution region was mainly around the eastern Qinghai–Tibet Plateau.

**Table 2:** Areas and percentages of habitat suitability distributions for *Notopterygium incisum* in different provinces and autonomous regions under the current environment

Region	Percentage of area (%)			Area (10^3^ km^2^)		
	Suitable habitat	Marginally suitable habitat	Unsuitable habitat	Suitable habitat	Marginally suitable habitat	Unsuitable habitat
Gansu	4.20	4.31	91.49	19.06	19.55	415.09
Qinghai	1.93	1.82	96.25	13.86	13.07	691.34
Tibet	1.01	2.23	96.76	11.69	25.82	1,120.17
Sichuan	8.02	7.85	84.13	38.71	37.89	406.12
Shaanxi	0.12	0.90	98.98	0.25	1.89	207.54
Other area				0.19	4.5	

### 3.3 Suitable habitat distributions under climate change

Currently, the suitable habitat for *N. incisum* is approximately 0.78% of the land area in China. According to the forecasted results of the EM, the area of suitable habitats of *N. incisum* showed a decreasing trend. The area percentages were 0.65% in the 2050s (average of four RCPs results) and 0.60% in the 2070s (average of four RCPs results). From the present to 2070, the areas of suitable habitat for *N. incisum* in western Sichuan Province and northeastern Tibet Autonomous Region will be significantly reduced. In Chongqing municipality and Hubei Province, the areas of suitable habitat increased, but the distributions became more fragmented. In addition, many of the suitable habitats turned into marginally suitable habitats (Figures 4 and 5). The area of suitable habitats decreased gradually with habitat fragmentation in all RCPs scenarios in the future. The marginally suitable habitats decreased in area. Marginally suitable habitats showed an increasing trend by the 2050s but showed a decreasing trend by the 2070s. In general, the suitable habitats for *N. incisum* are currently limited in area, and as the effects of climate change become more serious, a large part of the suitable habitat will turn into marginally suitable habitat or unsuitable habitat. Only a few unsuitable habitat areas advanced to marginally suitable habitats or suitable habitats, and the areas of marginally suitable habitats only occasionally became suitable habitats. In brief, *N. incisum* habitats are declining. Hence, saving the habitat resources for *N. incisum* and restoring its habitat are urgent tasks.

## 4 DISCUSSION

### 4.1 Model rationality

Currently, several studies have been devoted to identifying the current distribution range of *N. incisum* (Shang et al., 2015; Sun et al., 2015), but there are many shortcomings in these studies, and one of them is the use of a simple algorithm, which can delimit only the general distribution scope and cannot provide many landscape details (Sun et al., 2015). Other studies have focused on only the local distribution of this species (Shang et al., 2015). In contrast to previous studies, we used an EM based on nine different models with different mathematical algorithms to simulate the potential distribution of *N. incisum* throughout China. This model strategy avoids the selection of a single best model, thus eliminating (or at least limiting) model selection bias and, even more importantly, reducing the uncertainty of the modelling results caused by different modelling algorithms (Guisan et al., 2017; Guo et al., 2019); additionally, this strategy can provide significant landscape details and show the true distribution characteristics.

Environmental variables that can effectively and reliably describe the suitable habitat characteristics of the target species are the basic element in successful species distribution research. Bioclimatic variables are one of the most widely used environmental variables in SDM research. These variables describe climatic characteristics such as the average, extreme and seasonal, or annual changes in climate factors that have ecological significance (Fick & Hijmans, 2017; Title & Bemmels, 2018; Vega, Pertierra, & Olalla‐Tárraga, 2017). Here, based on PCA, the redundancy of bioclimatic variables was further reduced. Then, we selected a subset of bioclimatic variables for the model based on the characteristics of species habitats. These processes guarantee the accuracy of the model.

In nature, *N. incisum* is not a constructive or dominant species; thus, at the regional scale, the vegetation and soil conditions dictate the distribution of the species, even in a favorable climate. Thus, during the modelling process, the soil and vegetation types were used as overlying variables to further test the prediction results, and including these variables made the prediction results more realistic.

### 4.2 Habitat area estimates for *Notopterygium incisum*

According to previous studies, the optimal potential habitats for *N. incisum* in some parts of Sichuan Province, Tibet Autonomous Region, Qinghai Province, and Gansu Province have areas >1.42 × 10^5^ km^2^ (Sun et al., 2009). The most suitable habitat is in Sichuan Province, and more than 60% of the most suitable area for *N. incisum* is located in western Sichuan, such as in Aba and Ganzi prefectures (Sun et al., 2009, 2015). The EM results showed that the suitable habitats for *N. incisum* were mainly distributed in Sichuan Province, Gansu Province, Qinghai Province, Tibet Autonomous Region, and Shaanxi Province (Figure 3). In addition, the current suitable habitat area for *N. incisum* in mainland China covers 83.76 × 10^3^ km^2^, and the suitable habitats are mainly distributed in Sichuan Province, with an area of approximately 38.71 × 10^3^ km^2^, which is approximately 8.02% of the total area of Sichuan Province (Table 2). Our model result is consistent with that of previous studies in terms of the spatial range (Sun et al., 2009, 2015) but provides more details about the distribution of *N. incisum* habitats. The model results indicated that by the 2070s, the areas of suitable habitats will have persistently decreased (Figures 3, 4, 5). Moreover, the suitable habitats for *N. incisum* are narrowly distributed, and in the centralized distribution area, most suitable habitats will remain roughly unchanged in the future. This result suggests that the suitable habitats and marginally suitable habitats should be selected as resource conservation areas for the sustainable utilization of wild *N. incisum* resources. At the same time, suitable habitats should be taken into consideration when selecting and constructing the GAP base of *N. incisum.*

Our model results show that under global warming scenarios, the suitable habitat for *N. incisum* will continue to decrease with no obvious northward movement due to the dramatic topographic changes in the Qinghai–Tibet Plateau. At present, the *N. incisum* habitats are small and show noncontinuous, patchy, and mosaic distribution forms. Our model results show that climate change will intensify the destruction and fragmentation of native *N. incisum* habitats, which will lead to the disappearance of suitable habitat in these areas, increasing the risk of the extinction of this species.

### 4.3 The dominant environmental index response to suitability

**Figure 7:**
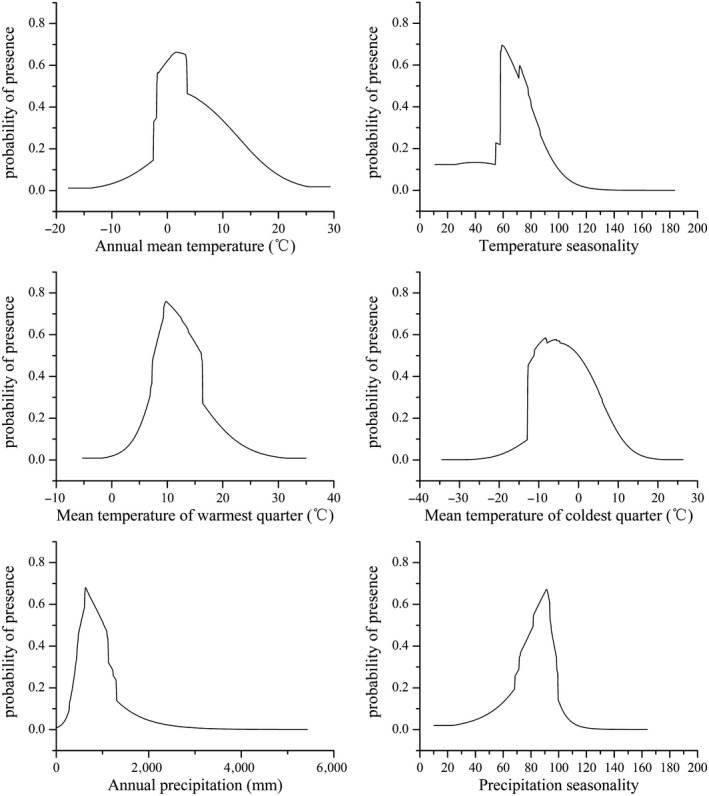
The dominant environmental index response curve. Annual mean temperature (bio1), temperature seasonality (bio4), mean temperature of warmest quarter (bio10), mean temperature of coldest quarter (bio11), annual precipitation (bio12), and precipitation seasonality (bio15)

Previous studies have shown that cold tolerance, growth‐season temperatures, and available water supply are the main environmental indexes that influence the distribution of alpine vegetation (Woodwand, 1987). In this study, according to the model results, the major variables that affected the distribution of *N. incisum* habitats were annual mean air temperature, temperature seasonality, mean temperature of warmest quarter, mean temperature of coldest quarter, annual precipitation, and precipitation seasonality. To further clarify the suitable ranges of environmental factors and the critical values, we drew the response curves for each variable mentioned above (Figure 7) and defined the suitable ranges of the variables (logistic probability of presence > 0.3). The results showed that the suitable ranges were −2.5 to 16.9°C for annual mean air temperature, 6.0–8.6°C for temperature seasonality, 6.8 –16.3°C for mean temperature of warmest quarter, −12.8 to 7.7°C for mean temperature of coldest quarter, 419–1181 mm for annual precipitation, and 71.4–98.5 mm for precipitation seasonality. The habitat characteristics of *N. incisum,* such as cold humid climates, were summarized through these environmental indexes. The results were more detailed than those of previous studies (Shang et al., 2015; Sun et al., 2015).

Climate change will influence *N. incisum* physiology through several interrelated processes, which will lead to changes in *N. incisum* distributions. As a perennial herb, freezing winter temperatures (i.e., bio11) at middle to high latitudes will harm the underground rhizome of *N. incisum*, which will be a main limiting factor affecting the *N. incisum* distribution; however, this situation may change in some areas with a warming climate. In addition, climate change will contribute to the increase in accumulated temperatures and lengthen the duration of plant growth (Zhang, Li, Ding, & Zheng, 2017). As a medicinal plant, the root and rhizome have been used in traditional Chinese medicine; hence, with increasing accumulated temperatures, the production of medicinal material will increase. Furthermore, in high and cold regions, increasing temperatures will lengthen the duration of soil moisture necessary for plant growth and reduce frost damage. However, if temperatures exceed their physiological optimum range, the growth and reproduction of *N. incisum* will be affected, and suitable habitats will gradually disappear.

Similarly, changes in precipitation patterns can influence the *N. incisum* distribution. Global warming will lead to an increase in precipitation in western China, especially the Qinghai–Tibet Plateau; hence, the water supply available for plants will increase. Nevertheless, prior work has suggested a relatively low impact of precipitation on the distribution of *N. incisum*; rather, the impact of temperature change will be the strongest (Shang et al., 2015; Sun et al., 2015). Indeed, in this study, nearly all 10 single models showed that among the nine environmental variables, all four temperature variables had key impacts on the distribution of *N. incisum*, and for the EM, the contribution rates of the temperature variables were higher than those of the precipitation variables, which indicated that the *N. incisum* distributions were less sensitive to precipitation variation than temperature variation.

## 5 CONCLUSIONS

Numerous studies have indicated that species will shift their geographic distributions poleward and upslope due to the dramatic topographic changes in the Qinghai–Tibet Plateau; this shift will be complex and heterogeneous. Here, we conducted an analysis of the endangered medicinal plant *N. incisum*, which is mainly distributed in the eastern margin of the Qinghai–Tibet Plateau, and we built a CHS model to simulate the distribution of *N. incisum*‐suitable habitats in three time periods (current, 2050s, and 2070s). The results showed that under climate change, the suitable habitats for *N. incisum* would significantly decrease with no obvious northward movement, and the increase in temperature would intensify the destruction and fragmentation of native habitats.

This study was a successful attempt to model the distribution of a medicinal plant with CHS and provided new information for modelling medicinal plant distributions in an area with complicated topography and a variable climate. We also tried to provide valuable insights to highlight the priority areas for medicinal plant conservation and resource utilization.

## CONFLICT OF INTEREST

The authors have no competing interests to declare.

## AUTHOR CONTRIBUTIONS

ZFZ, YLG, HYW, and WG conceived and designed the study. ZFZ and YLG collected the samples and established the model. ZFZ, QR, JL, and QZZ analyzed the data. ZFZ, YLG, HYW, and WG wrote the paper. All authors read and approved the final product.

## Data Availability

All environmental variables used in the manuscript are already publicly accessible, and we have provided the download address in the manuscript; relevant sampling site information can be found in Table S1 in the online version.

## References

[ece36117-bib-0001] Allouche, O. , Tsoar, A. , & Kadmon, R. (2006). Assessing the accuracy of species distribution models: Prevalence, kappa and the true skill statistic (TSS). Journal of Applied Ecology, 43(6), 1223–1232.

[ece36117-bib-0002] Anderson, R. P. (2013). A framework for using niche models to estimate impacts of climate change on species distributions. Annals of the New York Academy of Sciences, 1297, 8–28. 25098379 10.1111/nyas.12264

[ece36117-bib-0003] Araújo, M. B. , & New, M. (2007). Ensemble forecasting of species distributions. Trends in Ecology & Evolution, 22(1), 42–47. 17011070 10.1016/j.tree.2006.09.010

[ece36117-bib-0004] Barbet‐Massin, M. , Jiguet, F. , Albert, C. H. , & Thuiller, W. (2012). Selecting pseudo‐absences for species distribution models: How, where and how many? Methods in Ecology and Evolution, 3, 327–338.

[ece36117-bib-0005] Basile, M. , Valerio, F. , Balestrieri, R. , Posillico, M. , Bucci, R. , Altea, T. , … Matteucci, G. (2016). Patchiness of forest landscape can predict species distribution better than abundance: The case of a forest‐dwelling passerine, the short‐toed treecreeper, in central Italy. PeerJ, 4, e2398. 27651990 10.7717/peerj.2398 PMC5018664

[ece36117-bib-0006] Bell, D. M. , & Schlaepfer, D. R. (2016). On the dangers of model complexity without ecological justification in species distribution modeling. Ecological Modelling, 330, 50–59.

[ece36117-bib-0007] Booth, T. H. , Nix, H. A. , Busby, J. R. , & Hutchinson, M. F. (2014). Bioclim: The first species distribution modelling package, its early applications and relevance to most current maxent, studies. Diversity and Distributions, 20, 1–9.

[ece36117-bib-0008] Brown, J. L. , Bennett, J. R. , & French, C. M. (2017). SDMtoolbox 2.0: The next generation Python‐based GIS toolkit for landscape genetic, biogeographic and species distribution model analyses. PeerJ, 5, e4095. 29230356 10.7717/peerj.4095 PMC5721907

[ece36117-bib-0009] Committee of Flora of China (1986). Flora of China (pp. 190–191). Beijing, China: Science Press.

[ece36117-bib-0010] Committee of National Pharmacopoeia (2015). Pharmacopoeia of the People's Republic of China (pp. 182). Beijing, China: China Medical Science Press.

[ece36117-bib-0012] CVH (2019). CVH Home Page. Retrieved from http://www.cvh.ac.cn/

[ece36117-bib-0013] Elsen, P. R. , & Tingley, M. W. (2015). Global mountain topography and the fate of montane species under climate change. Nature Climate Change, 5(8), 772–776.

[ece36117-bib-0014] Fick, S. E. , & Hijmans, R. J. (2017). Worldclim 2: New 1‐km spatial resolution climate surfaces for global land areas. International Journal of Climatology, 37, 4302–4325.

[ece36117-bib-0015] Friedman, J. H. (1991). Multivariate adaptive regression splines. Annals of Statistics, 19, 1–67.

[ece36117-bib-0016] Gao, L. H. , & Fang, Z. S. (2007). Integrative analysis and evaluation of wild Rhizoma et *Radix Notopterygii* resource in Gansu. Pratacultural Science, 24, 11–14.

[ece36117-bib-0017] GBIF.org (2019). GBIF Home Page. Retrieved from https://www.gbif.org

[ece36117-bib-0018] Grenouillet, G. , Buisson, L. , Casajus, N. , & Lek, S. (2011). Ensemble modelling of species distribution: The effects of geographical and environmental ranges. Ecography, 34(1), 9–17.

[ece36117-bib-0019] Guisan, A. , Thuiller, W. , & Zimmermann, N. E. (2017). Habitat suitability and distribution models: With applications in R. Cambridge, UK: Cambridge University Press.

[ece36117-bib-0020] Guo, Y. L. , Li, X. , Zhao, Z. F. , & Nawaz, Z. (2019). Predicting the impacts of climate change, soils and vegetation types on the geographic distribution of *Polyporus umbellatus* in China. Science of the Total Environment, 648, 1–11. 30103037 10.1016/j.scitotenv.2018.07.465

[ece36117-bib-0021] Guo, Y. L. , Li, X. , Zhao, Z. F. , & Wei, H. Y. (2018). Modeling the distribution of *Populus euphratica* in the Heihe River Basin, an inland river basin in an arid region of China. Science China‐Earth Sciences, 61, 1669–1684.

[ece36117-bib-0023] IPCC (2013). Climate change 2013: The physical science basis, Contribution of Working Group I to the fifth assessment report of the Intergovernmental Panel on climate change. Cambridge, UK: Cambridge University Press.

[ece36117-bib-0024] Jiang, S. Y. , Sun, H. , Wu, X. C. , Zhou, Y. , Ma, X. J. , & Wu, R. (2006). Analysis and quality assessment standard of heavy metals and arsenic in Rhizoma et radix *Notopterygii* from different localities. China Journal of Chinese Materia Medica, 31, 978–980. 17048642

[ece36117-bib-0025] Jones, C. C. , Acker, S. A. , & Halpern, C. B. (2010). Combining local‐and large‐scale models to predict the distributions of invasive plant species. Ecological Applications, 20(2), 311–326. 20405790 10.1890/08-2261.1

[ece36117-bib-0026] Li, C. , Zhou, G. , & Zhou, Y. (2011). Determination the contents of six trace elements in different organs of wild *Notopterygium incisum* Ting ex H. T. Chang and *Notopterygium forbesii* Boiss by flame atomic absorption spectrometry. Chinese Journal of Pharmaceutical Analysis, 31, 1880–1883.

[ece36117-bib-0027] Li, X. , Nan, Z. T. , Chen, G. D. , Ding, Y. J. , Wu, L. Z. , Wang, L. X. , … Zhu, Z. M. (2011). Toward an improved data stewardship and service for environmental and ecological science data in west China. International Journal of Digital Earth, 4, 347–359.

[ece36117-bib-0028] Li, X. , Tian, H. , Wang, Y. , Li, R. , Song, Z. , Zhang, F. , … Li, D. (2013). Vulnerability of 208 endemic or endangered species in china to the effects of climate change. Regional Environmental Change, 13, 843–852.

[ece36117-bib-0029] Li, X. , & Wang, Y. (2013). Applying various algorithms for species distribution modelling. Integrative Zoology, 8(2), 124–135. 23731809 10.1111/1749-4877.12000

[ece36117-bib-0030] Li, Z. Y. , Zhang, X. S. , Wang, J. L. , & Lei, G. L. (2003). Progress in researches on *Notopterygium* root. Journal of Shaanxi College of Traditional Chinese Medicine, 26, 56–59.

[ece36117-bib-0031] Liu, Q. (2006). Studies on the XXXentia rulesand major influence factors of two endangered resource plant *NdtPoetgrium* spp. Master thesis, Sichuan University, China.

[ece36117-bib-0032] Lopatin, J. , Dolos, K. , Hernández, H. J. , Galleguillos, M. , & Fassnacht, F. E. (2016). Comparing generalized linear models and random forest to model vascular plant species richness using LiDAR data in a natural forest in central Chile. Remote Sensing of Environment, 173, 200–210.

[ece36117-bib-0033] Marzloff, M. P. , Oliver, E. C. J. , Barrett, N. S. , Holbrook, N. J. , James, L. , Wotherspoon, S. J. , & Johnson, C. R. (2018). Differential vulnerability to climate change yields novel deep‐reef communities. Nature Climate Change, 8(10), 873–878.

[ece36117-bib-0034] Mcquillan, M. A. , & Rice, A. M. (2015). Differential effects of climate and species interactions on range limits at a hybrid zone: Potential direct and indirect impacts of climate change. Ecology and Evolution, 5, 5120–5137. 26640687 10.1002/ece3.1774 PMC4662315

[ece36117-bib-0035] Mi, C. , Huettmann, F. , Guo, Y. , Han, X. , & Wen, L. (2017). Why choose Random Forest to predict rare species distribution with few samples in large undersampled areas? Three Asian crane species models provide supporting evidence. PeerJ, 5, e2849. 28097060 10.7717/peerj.2849 PMC5237372

[ece36117-bib-0036] Moisen, G. G. , Freeman, E. A. , Blackard, J. A. , Frescino, T. S. , Zimmermann, N. E. , & Edwards, T. C. Jr (2006). Predicting tree species presence and basal area in Utah: A comparison of stochastic gradient boosting, generalized additive models, and tree‐based methods. Ecological Modelling, 199, 176–187.

[ece36117-bib-0037] Muñoz‐Mas, R. , Papadaki, C. , Martinez‐Capel, F. , Zogaris, S. , & Ntoanidis, L. (2016). Generalized additive and fuzzy models in environmental flow assessment: A comparison employing the West Balkan trout (Salmo farioides; Karaman, 1938). Ecological Engineering, 91, 365–377.

[ece36117-bib-0038] Myan, F. W. Y. , Walker, J. , & Paramor, O. (2013). The interaction of marine fouling organisms with topography of varied scale and geometry: A review. Biointerphases, 8(1), 1–13. 24706140 10.1186/1559-4106-8-30

[ece36117-bib-0040] NTPDC (2019). Retrieved from http://data.tpdc.ac.cn

[ece36117-bib-0041] Pacifici, M. , Foden, W. B. , Visconti, P. , Watson, J. E. M. , Butchart, S. H. M. , Kovacs, K. M. , … Rondinini, C. (2015). Assessing species vulnerability to climate change. Nature Climate Change, 5, 215–225.

[ece36117-bib-0042] Patsiou, T. S. , Conti, E. , Zimmermann, N. E. , Theodoridis, S. , & Randin, C. F. (2015). Topo‐climatic microrefugia explain the persistence of a rare endemic plant in the alps during the last 21 millennia. Global Change Biology, 20(7), 2286–2300. 10.1111/gcb.12515 24375923

[ece36117-bib-0043] Peng, X. H. , Dong, S. J. , Zhu, H. W. , & Yan, F. (2006). The survey *Notopterygium* resources. Gansu Agricultural, 6, 135–135.

[ece36117-bib-0044] Phillips, S. J. , Anderson, R. P. , & Schapire, R. E. (2006). Maximum entropy XXXentianac of species geographic distributions. Ecological Modelling, 190, 231–259.

[ece36117-bib-0045] R Project (2018). The R project for statistical computing. Retrieved from https://www.r-project.org/

[ece36117-bib-0046] Ran, Y. H. , Li, X. , Lu, L. , & Li, Z. Y. (2012). Large‐scale land cover mapping with the integration of multi‐source information based on the Dempster‐Shafer theory. International Journal of Geographical Information Science, 26, 169–191.

[ece36117-bib-0048] Segurado, P. , & Araujo, M. B. (2004). An evaluation of methods for modelling species distributions. Journal of Biogeography, 31, 1555–1568.

[ece36117-bib-0049] Shang, X. , Dong, L. J. , Wen, L. J. , Peng, W. F. , Xu, X. L. , & Fang, Q. M. (2015). Study on suitable distribution areas of *Notopterygium incisum* in Sichuan province based on remote sensing and GIS. China Journal of Chinese Materia Medica, 40, 2553–2558. 26697677

[ece36117-bib-0050] Sun, H. , Jiang, Y. Y. , Chen, S. L. , Zhou, Y. , Xie, C. Y. , Ma, X. J. , & Chen, T. Z. (2009). Studies on habitats suitability of endangered medicinal plant *Notopterygium incisum* . China Journal of Chinese Materia Medica, 34, 535–538. 19526777

[ece36117-bib-0051] Sun, H. B. , Sun, H. , Jiang, S. Y. , Zhou, Y. , Cao, W. L. , Ji, M. C. , … Yan, H. J. (2015). Cultural regionalization for *Notopterygium incisum* based on 3S technology platform I. Evaluation for growth suitability for *N. ncisum* based on ecological factors analysis by Maxent and ArcGIS model. China Journal of Chinese Materia Medica., 40, 853–862. 26087545

[ece36117-bib-0052] Thuiller, W. , Lafourcade, B. , Engler, R. , & Miguel, B. A. (2009). BIOMOD‐a platform for ensemble forecasting of species distributions. Ecography, 32, 369–373.

[ece36117-bib-0053] Thuiller, W. , & Lavorel, S. (2010). Generalized models vs. classification tree analysis: Predicting spatial distributions of plant species at different scales. Journal of Vegetation Science, 14, 669–680.

[ece36117-bib-0054] Title, P. O. , & Bemmels, J. B. (2018). ENVIREM: An expanded set of bioclimatic and topographic variables increases flexibility and improves performance of ecological niche modeling. Ecography, 41, 291–307.

[ece36117-bib-0055] Urban, M. C. (2015). Accelerating extinction risk from climate change. Science, 348, 571–573. 25931559 10.1126/science.aaa4984

[ece36117-bib-0056] Vega, G. C. , Pertierra, L. R. , & Olalla‐Tárraga, M. (2017). MERRAclim, a high‐resolution global dataset of remotely sensed bioclimatic variables for ecological modelling. Scientific Data, 4, 170078. 28632236 10.1038/sdata.2017.78 PMC5477563

[ece36117-bib-0058] Woodwand, F. I. (1987). Climate and plant distribution. Cambridge, UK: Cambridge University Press.

[ece36117-bib-0059] WorldClim (2019). WorldClim Version2. Retrieved from http://worldclim.org/version2

[ece36117-bib-0060] Wu, D. , Zhao, X. , Liang, S. , Zhou, T. , Huang, K. , Tang, B. , & Zhao, W. (2015). Time‐lag effects of global vegetation responses to climate change. Global Change Biology, 21(9), 3520–3531. 25858027 10.1111/gcb.12945

[ece36117-bib-0061] Yang, L. C. , Zhou, G. Y. , Li, C. L. , Song, W. Z. , & Chen, G. C. (2011). Potetial refugia in Qinghai‐Tibetan plateau revealed by the phylogeographical study of *Swertia tetraptera* (Gentianaceae). Polish Journal of Ecology, 59, 753–764.

[ece36117-bib-0062] Zhang, D. , Fengquan, L. , & Jianmin, B. (2000). Eco‐environmental effects of the Qinghai‐Tibet Plateau uplift during the Quaternary in China. Environmental Geology, 39, 1352–1358.

[ece36117-bib-0063] Zhang, L. , Liu, S. , Sun, P. , Wang, T. , Wang, G. , Wang, L. , & Zhang, X. (2016). Using DEM to predict *Abies faxoniana* and *Quercus aquifolioides* distributions in the upstream catchment basin of the Min River in southwest China. Ecological Indicators, 69, 91–99.

[ece36117-bib-0064] Zhang, M. G. , Zhou, Z. K. , Chen, W. Y. , Cannon, C. H. , Raes, N. , & Slik, J. W. F. (2014). Major declines of woody plant species ranges under climate change in Yunnan, China. Diversity & Distributions, 20, 405–415.

[ece36117-bib-0065] Zhang, Y. L. , Li, L. H. , Ding, M. J. , & Zheng, D. (2017). Greening of the Tibetan plateau and its drivers since 2000. Chinese Journal of Nature, 39, 173–178.

[ece36117-bib-0066] Zhao, Z. F. , Wei, H. Y. , Guo, Y. L. , & Gu, W. (2016). Potential distribution of *Panax ginseng* and its predicted responses to climate change. Chinese Journal of Applied Ecology, 27, 3607–3615. 29696859 10.13287/j.1001-9332.201611.040

[ece36117-bib-0067] Zhou, Y. , Jiang, S. Y. , Ma, X. J. , Sun, H. , Pu, F. D. , & Wu, R. (2003). Resource crisis and protective measures on Rhizoma et Radix *Notopterygii* . Chinese Traditional & Herbal Drugs, 34, 382–384.

