# Supplementary material for: Potential distribution of Notopterygium incisum Ting ex H. T. Chang and its predicted responses to climate change based on a comprehensive habitat suitability model
Source: Ecol Evol. 2020 Mar 5;10(6):3004–16. doi: 10.1002/ece3.6117 (PMC7083672; doi:10.1002/ece3.6117)
Supplement: Supplementary file 1 [file ECE3-10-3004-s001.doc]

**Supplementary materials 1**

The principal component analysis (PCA) process to select a subset of the environmental variables

In this study, we adopted a PCA strategy proposed by Guisan et al (2017) to visualize the correlation between the variables and identify the main environmental gradients in the region to be used in the modeling process.Under this strategy, we performed a PCA to visualize the correlations between the variables and to identify the main environmental gradients in the region to be used in the modeling process.

First, we used all 19 bioclimatic variables to perform the PCA and only retained two principal component axes to summarize the whole environmental space (Fig S1-b).

Second, we investigated the distribution of *Notopterygium incisum* in the environmental space defined by the PCA.


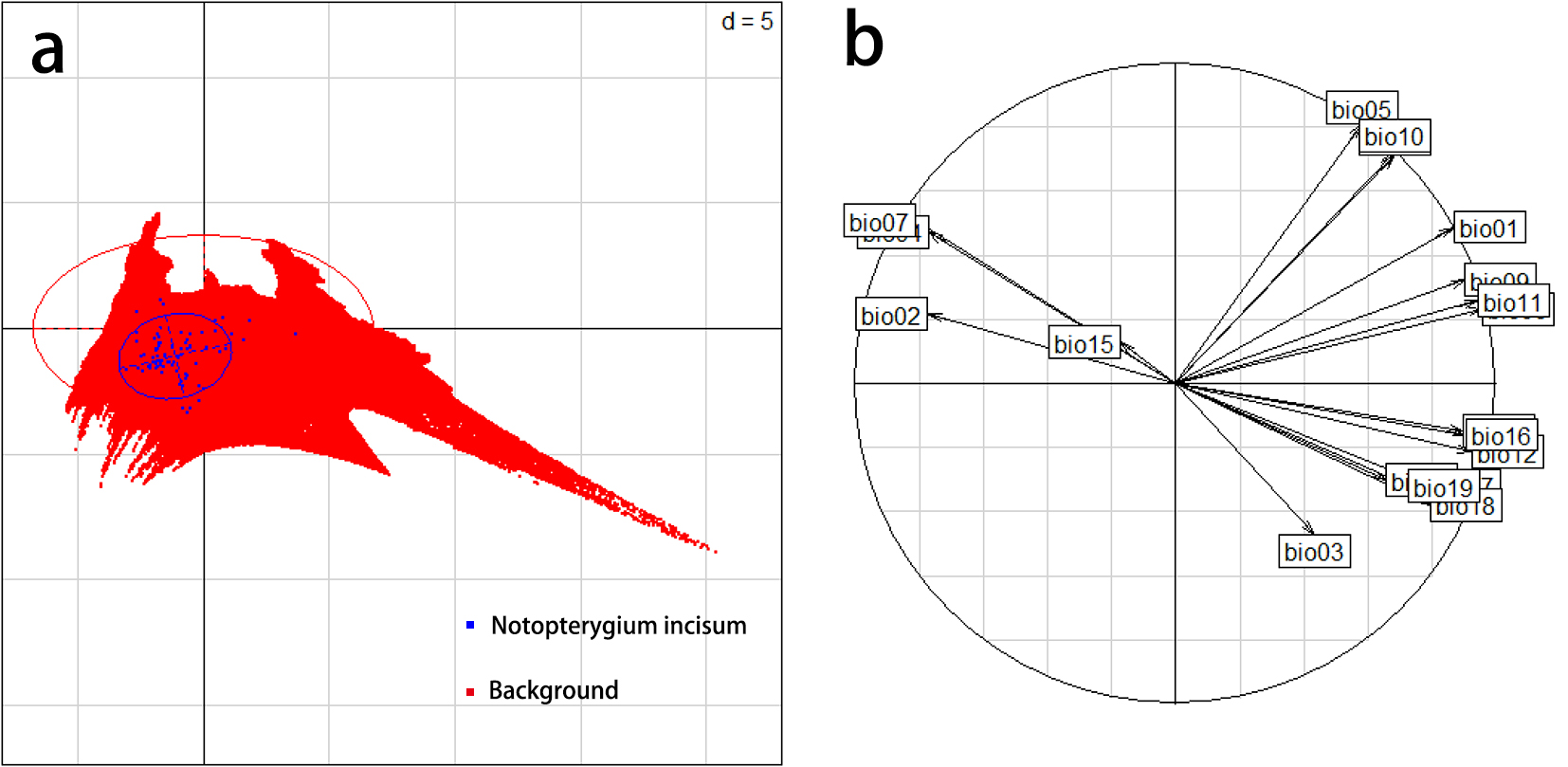


Figure S1 Distribution of the points of *Notopterygium incisum* occurrence in the environmental space defined by the first two PCA axes (a) and the correlation circle of the selected bioclimatic variables as a function of these first two PCA axes (b).Figure S1-a: Illustration of the distribution of *Notopterygium incisum* along the first two PCA axes. Figure S1-b: Illustration of the projection of the selected bioclimatic variables over the same two PCA axes.

Third, according to Figure S1-b, we could identify those variables presenting significant collinearity (i.e., two variables pointing in orthogonal directions are independent, and two variables pointing in the same or opposite directions are highly dependent).

Fourth, referring to Figure S1, we noted that the longest axis of the species’ ellipse (*Notopterygium incisum*) in Figure S1-a broadly follows the axis of the Bio11 variable. Thus, Bio11 is a good variable for discriminating between our species occurrences and the rest of the environment.

Fifth, according to the ecological characteristics of the target species and Figure S1-b, we choose six other bioclimatic variables: annual mean temperature, bio1; temperature seasonality, bio4; mean temperature of the warmest quarter, bio10; mean temperature of the coldest quarter, bio12; precipitation seasonality, bio15; precipitation of the coldest quarter, bio19

The code for this process can be downloaded from https://github.com/vdicolab/hsdm/blob/master/Part_6.Rmd

Reference

Guisan, A., Thuiller, W., Zimmermann, N.E., 2017. Habitat Suitability and Distribution Models: With Applications in R. Cambridge University Press, Cambridge
